# Autoimmune Pulmonary Alveolar Proteinosis: A Rare Diagnosis in Pediatric Age

**DOI:** 10.1177/23247096251323188

**Published:** 2025-03-14

**Authors:** Inês Pais-Cunha, Augusta Gonçalves, Sara Paulino, José Fontoura Matias, Silva Sónia, Catarina Ferraz, Inês Azevedo

**Affiliations:** 1 Serviço de Pediatria, UAG da Mulher e da Criança, ULS São João, Porto, PortugalULS São João, Porto, Portugal; 2 Faculdade de Medicina da Universidade do Porto, Porto, Portugal; 3 Serviço de Pediatria, ULS Braga, Braga, Portugal; 4 Unidade de Pneumologia Pediátrica, UAG da Mulher e da Criança, ULS São João, Porto, Portugal

**Keywords:** pulmonary alveolar proteinosis, autoimmunity, radiography

## Abstract

Autoimmune pulmonary alveolar proteinosis (AI-PAP) is a rare condition, especially in children. The clinical presentation ranges from asymptomatic forms to respiratory distress requiring ventilation. We describe the case of a 13-year-old adolescent male who presented to the emergency department with acute pleuritic chest pain not associated with systemic complaints. On examination, he had diminished breath sounds in the lower two thirds of the chest with no other abnormal findings; SpO_2_ (oxygen saturation) was 98% on room air. Chest radiograph revealed a marked interstitial infiltrate, comparable with the one taken 4 years earlier during an acute illness that was presumptively treated with azithromycin. A computed tomography (CT) scan revealed multiple bilateral areas of ground-glass opacities with areas of crazy paving, involving > 65% of lung parenchyma, suggestive of pulmonary alveolar proteinosis (PAP). Respiratory viral testing, including for coronavirus (SARS-CoV2), was negative. Bronchoalveolar lavage performed in the outpatient setting revealed a milky fluid and positive periodic acid-Schiff staining. Spirometry indicated a mild restrictive pattern (forced vital capacity [FVC] = 77%) and diffusing capacity of the lungs for carbon monoxide (DLCO) showed a moderate decrease at 48.6%. No mutations associated with surfactant dysfunction were found on the genetic panel. Anti-granulocyte macrophage colony-stimulating factor (GM-CSF) antibody testing was strongly positive, raising suspicion for autoimmune PAP. At 20 months of follow-up, the patient remains asymptomatic with a normal spirometry. Although treatment with agents, such as the inhaled form of granulocyte-macrophage colony-stimulating factor (GM-CSF) appears promising for the treatment of symptomatic adult patients, as this patient remains asymptomatic, a conservative approach was taken, and he continues to be monitored in the clinic.

## Introduction

Pulmonary alveolar proteinosis (PAP) is a rare condition with an overall prevalence of around 7 in 1 000 000 in the general population, increasing with age, although this is likely to be an underestimate given the diagnostic challenges underlying the condition.^1^ This group of conditions is characterized by the accumulation of surfactant in the alveoli, which can be caused by reduced clearance or abnormal production.^2^

Pulmonary surfactant plays a critical role in reducing alveolar surface tension, preventing alveolar collapse during breathing, and defending the lung against microbial pathogens. Surfactant clearance occurs primarily in type II alveolar epithelial cells or through uptake and removal by alveolar macrophages.^3^ Granulocyte-macrophage colony-stimulating factor (GM-CSF) is a cytokine essential for the maturation and function of alveolar macrophages.^4^

In primary PAP, GM-CSF signaling is disrupted, leading to alveolar macrophage dysfunction and impaired surfactant clearance.^5^ This may be the result of mutations affecting GM-CSF receptor expression (hereditary PAP) or of autoantibodies against GM-CSF (autoimmune PAP [AI-PAP]). In both cases, there is defective surfactant clearance by alveolar macrophages, which results in its accumulation.^6^ Secondary PAP arises from underlying conditions, such as hematological malignancies or chronic infections that reduce the number or function of alveolar macrophages.^5^ In addition, mutations in genes encoding surfactant proteins or in those involved in surfactant production and lung development can cause defective surfactant synthesis, contributing to its accumulation in the alveoli.^6^ Depending on the underlying etiology and age of onset, patients have a variable clinical presentation, ranging from asymptomatic forms in older patients to respiratory distress with the urgent need for intubation, typically in newborns.^7,8^

## Case Description

A 13-year-old adolescent male, with no significant previous medical history, presented to the emergency department with a 3-day history of acute bilateral pleuritic chest pain associated with mild non-productive cough and no dyspnea. Associated with this, he had mild rhinorrhea and a single febrile episode that day (temperature of 38ºC). Chest pain was localized to the costal margin region and worsened with cough, without diurnal variation. The patient-reported relief with paracetamol. There were no complaints of joint pain, weight loss, anorexia, fatigue, episodes of syncope or exercise restriction. In fact, he practiced sports regularly—canoeing 2 times a week. No evidence of an infectious exposure or contact with household or environmental fumes, dust, or mineral oils was described. There was no known family history of cardiopulmonary conditions. He had a chest radiograph taken 4 years earlier during an acute illness, which showed a marked interstitial infiltrate that was presumptively treated with azithromycin with no further clinical symptoms and no further follow-up.

**Figure 1.**
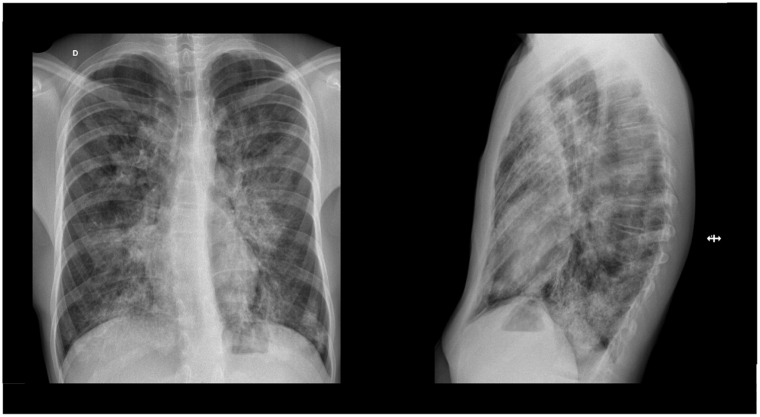
Chest radiograph showing marked bilateral interstitial infiltrate.

**Figure 2.**
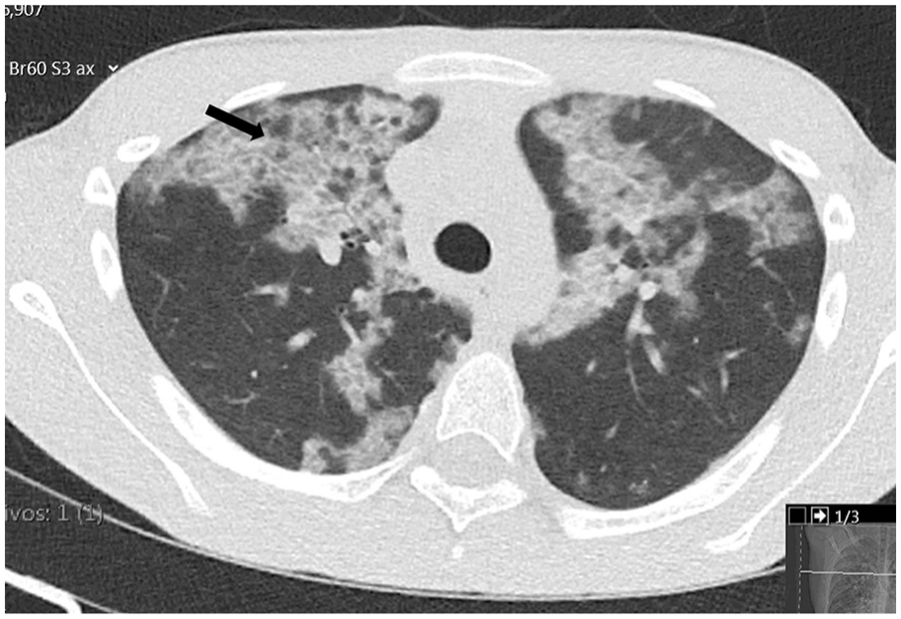
Chest CT showing multiple bilateral areas of ground-glass opacities involving > 65% of lung parenchyma with areas of crazy paving (arrow).

On admission, the patient’s temperature was 37.8°C with normal peripheral oxygen saturation (99%) in room air. His heart (93 beats per minute) and respiratory rate (15 breaths per minute) were normal and blood pressure was on the 85th percentile (115/66 mmHg). Physical examination revealed diminished breath sounds in the lower two thirds of the chest with no adventitious sounds. No respiratory distress, finger clubbing, cyanosis, abnormal heart sounds, or other findings were present. Chest radiograph revealed a marked interstitial infiltrate, comparable with his previous examination (Figure 1). A thoracic computed tomography (CT) revealed multiple bilateral areas of ground-glass opacities involving > 65% of lung parenchyma, suggestive of PAP (Figure 2). Respiratory viral testing was negative, and he remained stable throughout his monitoring in the emergency department. He was discharged with empiric antibiotics (amoxicillin-clavulanic acid and azithromycin) to cover a potential respiratory infection, with clinical resolution of symptoms and was sent for follow-up at the pediatric respiratory clinic.

Upon further investigation in the outpatient setting, positive antinuclear antibodies (ANAs) at a titer of 1/89 with a fine speckled pattern were detected, while other autoantibodies tested negative and immunoglobulin levels remained within normal limits. Bronchoalveolar lavage revealed fluid with a milky appearance and positive periodic acid-Schiff staining; microbiological examination, including for mycobacteria, returned negative results. Spirometry indicated a mild restrictive pattern with reduced forced vital capacity (FVC) at 2.92 L (77%) and forced expiratory volume in 1 second (FEV1) at 3.21 L (69.9%), alongside a normal FEV1/FVC ratio (109%). In addition, the DLCO single breath (SB) showed a moderate decrease at 13.8 ml/min/mmHg (48.6%). Suspecting PAP, a genetic panel was conducted, which showed no mutations associated with surfactant dysfunction. Subsequently, GM-CSF antibody testing was performed with a positive result, raising suspicion for AI-PAP. At 20 months of follow-up, the patient remains asymptomatic and continues to exercise regularly. He repeated spirometry testing with normal FVC at 4.03 L (81.3%); FEV1 at 3.71 L (87.5%); FEV1/FVC ratio at 91.96% and DLCO SB at 25.54 ml/min/mmHg (83.7%). As the patient remains stable with no respiratory symptoms, we decided to defer treatment and continue monitoring with regular clinic visits.

## Discussion

We present a rare case of AI-PAP incidentally diagnosed in an adolescent patient during an acute respiratory infection. The patient experienced improvement of respiratory symptoms shortly after discharge from the emergency department, despite no specific treatment for the underlying AI-PAP. He has remained asymptomatic since.

Although AI-PAP accounts for approximately 90% of PAP cases, its incidence in the pediatric population is minimal.^9^ Disease onset is still poorly described as patients usually present with symptoms after substantial alveolar filling has occurred.^10^ In fact, up to one third of patients with AI-PAP are asymptomatic, making this an even more challenging diagnosis.^11^

In our patient, the changes observed in the chest radiograph, consistent with a prior examination, prompted further investigation that led to the final diagnosis. This case underlines the importance of investigating children with persistent imaging changes as well as significant imaging changes that are not consistent with the patient’s clinical condition, as they may indicate an underlying pulmonary disease. In addition, a notable clinical feature of AI-PAP is the potential discrepancy between the severity of chest CT findings and the patients’ symptoms.^12^ In this case, despite marked abnormalities in the chest CT, the patient remained asymptomatic post-resolving acute infectious symptoms and was even able to maintain regular exercise routines. This was may be due to compensatory mechanisms, such as hypoxic pulmonary vasoconstriction, which reduces perfusion to areas of the lung with reduced ventilation and redirects blood to well-ventilated areas of the lung, maintaining ventilation-perfusion matching.^13,14^

The natural history of AI-PAP usually is variable and comprises three patterns: spontaneous improvement (in up to 7% of patients); stable symptomatic disease or progressive deterioration with respiratory failure.^9,10,15^ However, the clinical course in pediatric patients is still not well described in literature.

Although some patients, as in our clinical case, are asymptomatic, we should be aware of the pathophysiology of this disease to prevent and predict potential complications. Surfactant accumulation in PAP impairs gas exchange, causes alveolar infiltrates, and may reduce total lung capacity.^6^ Toxic proteins accumulating in type II pneumocytes further damage the cells.^11^ Pulmonary fibrosis, although rare, can develop in advanced disease.^5^ Patients may have normal pulmonary function tests or reduced FVC and reduced FEV1 (but normal or high FEV1/FVC ratio), indicating a restrictive lung pattern.^7^ The most common finding is a decreased DLCO due to impaired oxygen diffusion across the alveolar-capillary membrane, leading to an increased alveolar-arterial oxygen gradient.^6^ Furthermore, individuals with PAP are at significantly increased risk of secondary microbial infections.^5,6^

There is no rigorous evidence base for treatment of children with PAP and the results are sometimes not satisfying.^7^ A recent study by Griese et al, described 4 cases of pediatric AI-PAP with different symptomatic presentations that underwent different treatments. This included isolated inhaled GM-CSF therapy in a patient presenting with prolonged dyspnea but no indication of further systemic compromise, and whole lung lavage (WLL) with or without inhaled GM-CSF in 2 patients with progressive dyspnea and weight loss. There was 1 patient with progressive dyspnea, very low body weight and no response to first-line treatment with multiple WLL, who was started on plasmapheresis sessions and rituximab.^16^ Whole lung lavage, through the removal the accumulated surfactant, is reported to be efficient in managing symptomatic pediatric patients.^17^ Nevertheless, this is an invasive procedure with inherent risks and challenges, especially in children, which may not always guarantee long-term success.^18,19^ Alternatively, the administration of inhaled or subcutaneous recombinant GM-CSF, that acts through the competitive binding and neutralization of the disease-causing endogenous autoantibodies, has shown promising results.^20,21^ The inhaled form of GM-CSF seems to be the most effective^22^ and has been used as an augmentation therapy for patients with unsatisfactory results to WLL or as monotherapy.^23,24^ Although this evidence is mainly in the adult population, the aforementioned study in adolescents showed good response to monotherapy with inhaled GM-CSF in this age group.^16^ Furthermore, this seems to be the least invasive and most comfortable approach for young children.^16^ It is important to note that this case series refers to symptomatic patients and that the most recent guidelines from the European Respiratory Society only indicate treatment in active or worsening disease.^25^ In fact, a conservative approach is considered appropriate in asymptomatic patients.^8,26^

As there is still a lack of evidence in pediatric PAP, it is important to tailor our management options to our patients, choosing the one that seems to be more effective and with the best benefit/risk ratio, taking into account the resources available. In our patient, although he has an altered CT scan, he remains asymptomatic with no signs of respiratory distress or failure to thrive, so that, we decided to maintain clinical vigilance and defer pharmacological treatment. Close monitoring and early detection of signs of respiratory distress, hypoxia, and decline in lung function are essential to ensure timely treatment and prevent future deterioration.

In asymptomatic patients, we propose a regular follow-up with clinical visits and lung function tests including DLCO at 3- to 6-month intervals. Imaging studies may be useful for monitoring disease progression, but the optimal time to repeat a CT scan has not been established. It is important to carefully consider the risk-benefit ratio, particularly the potential risks of exposing children to radiation. We suggest that CT scanning may be considered in cases of clinical deterioration and when transitioning to adult care, but decisions should be tailored to each individual case. If the patient develops symptoms of respiratory distress, hypoxemia, or significant changes in lung function tests, treatment with exogenous GM-CSF and/or WLL should be considered. Refractory cases to first-line treatment, with progressing symptoms, requiring supplemental oxygen, may benefit from rituximab and, if this fails, plasmapheresis, although evidence remains scarce even in the adult population, and potential adverse events must be considered.^17^

In conclusion, PAP includes a group of conditions that lead to accumulation of proteinaceous material in the lungs that result in progressive dyspnea, hypoxemia, and risk of respiratory failure. Its identification should prompt detailed evaluation to determine the underlying cause. Although rare in the pediatric population, AI-PAP may be diagnosed in young patients, highlighting the importance of keeping this diagnosis in mind and requesting specific antibody studies. Detection of the underlying cause of PAP is essential for planning future management and treatment of these children.
